# Supplementary material for: Content-rich biological network constructed by mining PubMed abstracts
Source: BMC Bioinformatics. 2004 Oct 8;5:147. doi: 10.1186/1471-2105-5-147 (PMC528731; doi:10.1186/1471-2105-5-147)
Supplement: Additional File 2 — The original results of the above study (non-essential files are deleted to keep the file size under the limit set by BMC bioinformatics). [file 1471-2105-5-147-S2.bz2 › chilibotAdditionalFile2/dip05/367ID9045692E6041/html/FGF2_FGFR3.html]

 


 **FGF2** and **FGFR3** 
  
Found 77 abstracts in PubMed, retrieved 05.  
 

 What does Google say? 
 PDF only 
| .edu only 

---

**Interactive relationship** (e.g. stimulation, inhibition, etc)

**Neutral relationship**- The biological significance of VEGF, EGF, and bFGF  [ **FGF2** ]  is mediated by their receptors, which belong to the family of tyrosine kinase receptors Flt 1 VEGFR 1, KDR VEGFR 2, EGFR, FGFR 1, FGFR 2, FGFR 3  [ **FGFR3** ] , and FGFR 4.  Ref: 12500263 Microsc Res Tech, 2003

**Non-interactive relationship** (e.g. studied together, co-existance, homology, etc.)

- Using semiquantitative reverse transcription polymerase chain reaction, we examined the expression of insulin like growth factor 1 Igf 1, fibroblast growth factor 2  [ **FGF2** ]  Fgf 2  [ **FGF2** ] , and their receptors Igf 1r, Fgfr1, Fgfr2, and  **Fgfr3**  in the MCC of 28 day old rats at, and 14 days after placing intraoral appliances designed to produce a lateral functional shift of the mandible.  Ref: 12594422 Am J Orthod Dentofacial Orthop, 2003
